# From evidence to action: Understanding clinical practice guidelines

**DOI:** 10.1080/17453670902807458

**Published:** 2009-02-01

**Authors:** Rudolf W Poolman, Cees CPM Verheyen, Gino M Kerkhoffs, Mohit Bhandari, Holger J Schünemann

**Affiliations:** ^1^Department of Orthopedic Surgery, Onze Lieve Vrouwe GasthuisAmsterdamthe Netherlands; ^2^Department of Orthopaedic Surgery, Isala KliniekenZwollethe Netherlands; ^3^Department of Orthopedic Surgery, Orthotrauma Research Center, Academic Medical Center, University of Amsterdamthe Netherlands; ^4^Division of Orthopedic Surgery, McMaster UniversityOntarioCanada; ^5^Department of Epidemiology, Italian National Cancer Institute Regina ElenaRomeItaly

## Abstract

**ABSTRACT** Good guidelines will help us to take evidence into practice. In a survey among Dutch orthopedic surgeons, development and use of evidence-based guidelines was perceived as one of the best ways of moving from opinion-based to evidence-based orthopedic practice. The increasing number of guidelines means that knowing how to make a critical appraisal of guidelines is now a key part of every surgeon’s life. This is particularly true because guidelines use varying systems to judge the quality of evidence and the strength of recommendations. In this manuscript we discuss what a guideline is, where we can find guidelines, how to evaluate the quality of guidelines, and finally provide an example on the different steps of guideline development. Thus, we show that good guidelines are a summary of the best available evidence and that they provide a graded recommendation to help surgeons in evidence-based practice.

## Introduction

In the post-Second World War era, surgeons used a wide variety of treatment modalities for comparable conditions. The decisions for or against a certain treatment option were primarily based on the origin of the doctor’s training, and less frequently based on sound research findings (Mooij 1999). This inter-doctor variability was recognized by medical organizations and subsequently consensus meetings were conducted to develop clinical guidelines (Mooij 1999). Initially based on expert opinions without explicit evaluation of the available evidence, these consensus meetings later started to implement research findings in the consensus process (Mooij 1999).

Good guidelines will help us to take evidence into practice. In a survey among Dutch orthopedic surgeons, the development and use of evidence-based guidelines was perceived as one of the best ways to move from opinion-based to evidence-based orthopedic practice (Poolman et al. 2007b). However, the number of guidelines is increasing. For example, a search in the National Guideline Clearinghouse for guidelines on knee arthritis revealed 34 hits; a search on hip fracture guidelines resulted in 57 hits. This increasing number of guidelines requires that knowledge of how to make a critical appraisal of guidelines is now becoming a key part of every surgeon’s life. This is particularly true because guidelines use varying systems to judge the quality of evidence and the strength of recommendations. An increasing number of organizations have come to an agreement to use a common system for formulation of recommendations in guidelines. This system, the GRADE system, suggests that recommendations given in guidelines can be either strong or weak (Guyatt et al. 2008a). The quality of the underlying evidence is one of the factors that determines the strength of a recommendation. Thus, good guidelines summarize the best available evidence and provide a graded recommendation to help surgeons in evidence-based practice.

In this article, we provide principles of guideline development, and databases of guidelines, and we give a case example of the development of a recently developed set of guidelines.

### What are guidelines?

Guidelines are devised to help doctors in the clinical decision process. They should include a clear recommendation for action (Oxman et al. 2006, Guyatt et al. 2008a). In contrast to guidelines, systematic reviews provide no recommendations. Recommending a treatment is a key feature of a guideline. Guidelines are often mistaken for cookbooks; however, guideline is not just another word for law or rule. Although both should be followed and both can be acted upon, guidelines are less polished, more open to revision and alteration, and more likely to have exceptions in certain circumstances—depending on the strength of the recommendations. Furthermore, guidelines should be used as a resource. That is, relevant questions should be answered in a set of guidelines and not all recommendations necessarily apply to all patients. Any document is considered a guideline when it aims to streamline particular processes according to a set routine. By definition, following guidelines is never mandatory. A protocol would be a better term for a mandatory procedure. Guidelines can facilitate higher quality and predictability of action of healthcare workers.

In past centuries, consensus in medical decision making was reached through eminence-based strategies. Decision making was highly influenced by the simple fact that if an eminent professor stated something as a golden rule or a good idea, or guideline, then it was considered a guideline. The modern guideline-based approach to healthcare originated in the 1990s. In the Netherlands, guidelines are produced at the national level by medical associations. Two bodies, CBO (the Dutch Institute for Healthcare Improvement) and NHG (the Dutch College of General Practitioners) publish specialist and primary care guidelines, respectively.

Internationally, there has been progress in the development of guideline. Modern medical guidelines are documents designed to guide decisions and criteria in specific medical areas, as defined by the best research evidence available (Guyatt et al. 2008a). Clinical guidelines identify and evaluate the most current data about prevention, diagnosis and/or treatment, eventually including the dosage of medications, risk/benefit, and cost-effectiveness. Subsequently, these define the most important questions related to clinical practice and identify all possible decision options and their outcomes. Some guidelines contain decision algorithms to be followed. Other guidelines place the treatment alternatives into classes to help providers in deciding what treatment to use. An additional objective of a medical guideline is to achieve the best balance between cost and effectiveness; it has frequently been demonstrated that the use of guidelines in hospitals is an effective way to achieve best practice objectives (Goossens 2004).

### How can we find guidelines?

You can find many guideline resources on the internet. We suggest that one should start by searching for guidelines developed in one’s own country, as they are most suitable for local circumstances. Institutional guidelines are adjusted to the local situation, and are usually adapted from a professional organization’s guidelines or national guidelines. The National Guideline Clearinghouse (NGC) is a public resource for evidence-based clinical practice guidelines (http://www.guideline.gov/). It provides guidelines from several institutions, professional societies, and government agencies worldwide. A helpful feature is side-by-side comparison of guidelines. Another useful source is http://www.guidelines-international.net/. Furthermore, the TRIP database can be searched for EBM guidelines (http://www.tripdatabase.com/). In the UK, the National Institute for Health and Clinical Excellence (NICE) is the independent organization dedicated to guideline development (http://www.nice.org.uk/). Finally, the G-I-N (Guidelines International Network) created the International Guideline Library, which includes the International Guideline Database with over 5,000 guidelines. G-I-N is a global non-profit association of organizations and individuals involved in the development and use of clinical practice guidelines. It aims to improve the quality of healthcare by endorsing the systematic development of clinical practice guidelines and their application in practice, through worldwide collaboration (http://www.g-i-n.net).

### How can we evaluate the quality of guidelines?

The Grading of Recommendations Assessment, Development, and Evaluation (GRADE) approach is used by an increasing number of other organizations internationally (Schunemann et al. 2006b, Guyatt et al. 2008a, Guyatt et al. 2008b). In international meetings for over 8 years, the GRADE Working Group has developed a set of evidence-based criteria to grade the quality of evidence (Table 1) and the strength of recommendations (Table 2) (Schunemann et al. 2006b, Guyatt et al. 2008a). The GRADE system has several advantages over other systems, including explicit definitions and sequential judgments during the grading process; a detailed description of the criteria for the quality of evidence for single patient-important outcomes and for the overall quality of the evidence; weighing of the relative importance of outcomes; consideration of the balance between health benefits and harm, burdens, and cost; and the development of evidence profiles and summaries of findings. In addition, the GRADE group is supported by an international collaboration. The main limitation and criticism of the GRADE system is its complexity, but this degree of complexity is required to provide the necessary transparency in guideline development.

**Table 1.** Evidence-based criteria to grade the quality of evidence (Schunemann et al. 2006b)

Quality of evidence	Study design	Lower if **^a^**	Higher if **^a^**
High (4)	Randomized trial	Study limitations	Large effect
		– 1 serious	+ 1 large
		– 2 very serious	+ 2 very large
Moderate (3)		Inconsistency	Dose response
		– 1 serious	+ 1 evidence of a gradient
		– 2 very serious	
Low (2)	Observational study	Indirectness	All plausible confounding
		– 1 serious	
		– 2 very serious	+ 1 would reduce a demonstrated effect, or
Very low (1)		Imprecision	
		– 1 serious	+ 2 would suggest a spurious effect when results
		– 2 very serious	
		Publication bias	show no effect
		– 1 likely	
		– 2 very likely	

**^a^** 1 = move up or down one grade (for example from high to intermediate) 2 = move up or down two grades (for example from high to low)

**Table 2.** Determinants of strength of recommendation (Schunemann et al. 2006b)

Factors that can strengthen the strength of a recommendation	Comment
Balance between desirable and undesirable effects	The larger the difference between the desirable and undesirable consequences, the more likely a strong recommendation is warranted. The smaller the net benefit and the lower the certainty for that benefit, the more likely weak recommendations are warranted.
Quality of the evidence	The higher the quality of evidence, the more likely a strong recommendation.
Values and preferences	The greater the variability in values and preferences, or uncertainty in values and preferences, the more likely a weak recommendation is warranted.
Costs (resource allocation)	The higher the costs of an intervention—that is, the more resources consumed— the less likely a strong recommendation is warranted.

The AGREE (Appraisal of Guidelines for Research and Evaluation) collaboration (http://www.agreecollaboration.org/) endeavors to establish a shared framework for guideline development, reporting, and assessment. An instrument for the appraisal of clinical guidelines was developed through this collaboration (AGREE Collaboration, 2003). The AGREE instrument can be downloaded from the website and consists of six domains: (1) Scope and purpose deals with the overall aim of the guideline, the specific clinical questions, and the target patient population. (2) Stakeholder involvement focuses on the extent to which the guideline represents the views of its intended users. (3) Rigor of development relates to the process used to gather and synthesize the evidence, the methods to formulate the recommendations, and those to update them. (4) Clarity and presentation deals with the language and the format of the guideline. (5) Applicability pertains to the likely organizational, behavioral, and cost implications of applying the guideline. (6) Lastly, editorial independence is concerned with the independence of the recommendations and acknowledgement of possible conflicts of interests from the guideline development group (http://www.agreecollaboration.org/).

The Conference on Guideline Standardization (COGS) used a 2-stage modified Delphi process to develop standards for reporting clinical practice guidelines, which resulted in the COGS checklist (Table 3) (Shiffman et al. 2003, Oxman et al. 2006) (see http://gem.med.yale.edu/cogs/welcome.do). While many associations have their own typical formats for reporting guidelines, the COGS checklist is the only agreed standard for reporting guidelines between organizations (Oxman et al. 2006).

**Table 3.** The COGS checklist for reporting clinical practice guidelines (Shiffman et al. 2003) ^a^

Topic	Description
1. Overview material	Provide a structured abstract that includes the guideline’s release date, status (original, revised, updated), and print and electronic sources.
2. Focus	Describe the primary disease/condition and intervention/service/technology that the guideline addresses. Indicate any alternative preventive, diagnostic, or therapeutic interventions that were considered during development.
3. Goal	Describe the goal that following the guideline is expected to achieve, including the rationale for development of a guideline on this topic.
4. Users/setting	Describe the intended users of the guideline (e.g., provider types, patients) and the settings in which the guideline is intended to be used.
5. Target population	Describe the patient population eligible for guideline recommendations and list any exclusion criteria.
6. Developer	Identify the organization(s) responsible for development of the guideline and the names/credentials/potential conflicts of interest of individuals involved in the development.
7. Funding sources/sponsor	Identify the funding source/sponsor and describe its role in developing and/or reporting the guideline. Disclose potential conflicts of interests.
8. Evidence collection	Describe the methods used to search the scientific literature, including the range of dates and databases searched, and criteria applied to filter the evidence retrieved.
9. Recommendation grading criteria	Describe the criteria used to rate the quality of evidence that supports the recommendations, and the system for describing the strength of the recommendations. Recommendation strength communicates the importance of adherence to a recommendation and is based on both the quality of the evidence and the magnitude of anticipated benefits or harm.
10. Method for synthesizing evidence	Describe how evidence was used to create recommendations, e.g., evidence tables, meta-analysis, decision analysis.
11. Prerelease review	Describe how the guideline developer reviewed and/or tested the guidelines prior to release.
12. Update plan	State whether or not there is a plan to update the guideline and, if applicable, an expiration date for this version of the guideline.
13. Definitions	Define unfamiliar terms, and those that are critical to correct application of the guideline and that might be subject to misinterpretation.
14. Recommendations and rationale	State the recommended action precisely and the specific circumstances under which to perform it. Justify each recommendation by describing the linkage between the recommendation and its supporting evidence. Indicate the quality of evidence and the strength of recommendation, based on the criteria described in 9.
15. Potential benefits and harm	Describe anticipated benefits and potential risks associated with implementation of guideline recommendations.
16. Patient preferences	Describe the role of patient preferences when a recommendation involves a substantial element of personal choice or values.
17. Algorithm	Provide (where appropriate) a graphical description of the stages and decisions in clinical care described by the guide line.
18. Implementation considerations	Describe anticipated barriers to application of the recommendations. Provide references to any auxiliary documents for providers or patients that are intended to facilitate implementation. Suggest review criteria for measuring changes in care when the guideline is implemented.

**^a^** COGS: Conference on Guideline Standardization.

### Using guidelines to guide practice: a case example of guideline development

In the Netherlands, the organization facilitating the development of medical guidelines is CBO (the Dutch Institute for Healthcare Improvement), which is financed by the Order of Medical Specialists in collaboration with the Dutch College of General Practitioners. Since it was founded in 1979, more than 125 national guidelines have been published. The understanding is that every guideline should be revised after 5 years. The last Dutch guidelines “Deep Venous Thrombosis and Pulmonary Embolism” were published in 1999 and are due for revision, entitled “Diagnosis, Prevention and Treatment of Venous Thromboembolism and secondary prevention of Arterial Thrombosis”.

For the development of evidence-based guidelines, a sequence of steps were taken as illustrated in the Figure.

### Lack of high-quality evidence

Certain topics of the subject were ambiguous in the last guidelines because of lack of high-quality evidence or confusing recommendations. One example was the use of pharmacological thromboprophylaxis in daycare surgery (knee arthroscopy) or during plaster immobilization. Also, relevant questions from the professionals in the field had to be addressed.

When evidence is of low quality, recommendations will be weak. To make recommendations, a consensus will always have to be reached. Well-known methods for reaching a consensus are the Nominal Group Technique (NGT), the Delphi Method, and Consensus Conferences (Fretheim et al. 2006b).

### Fundamental questions and outline of text

Listings of major questions and dilemmas on the subject derived from the analysis of impediments were assembled. Also, a short set-up with planned chapters was proposed.

Guidelines can be helpful in clinical decision making, provided that they have been developed following rigorous methodology and provided that they address clinically and patient-important endpoints (Guyatt et al. 2008b). Thus, a guideline must start with a clearly focused clinical question. To identify endpoints that are important for patients, consumers (patients) can be made part of a consensus group. Consumer values may influence recommendations; thus, these recommendations should be marked as such in the final guidelines (Schunemann et al. 2006a).

**Figure:**
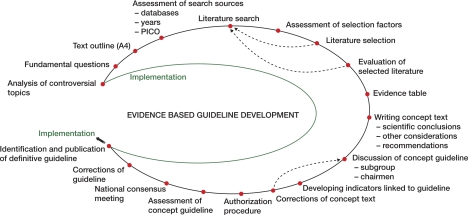
Evidence-based guideline development.

### Assessment of search sources

The main databases to be used were Medline/PubMed and Embase. The National Guidelines Clearinghouse and the Guidelines International Network were checked for existing guidelines on the subject. Furthermore, the Cochrane Database and the Database of Abstracts of Reviews of Effectiveness (DARE) were reviewed. With the AGREE instrument (Appraisal of Guidelines for Research and Evaluation) developed for the assessment of clinical practice guidelines, current guidelines were evaluated (AGREE Collaboration, 2003). Upon the results of this analysis the members of the VTE prevention subgroup decided to use the seventh conference on antithrombotic and thrombolytic therapy (ACCP) (2004) as a key reference for the new guidelines (Geerts et al. 2004) as well as the last Dutch guidelines on the subject. Database searches were concentrated from the time that ACCP ended until that particular day (2003–2006). When there was doubt or possible disagreement with the ACCP on certain issues, an extended search was conducted. In the PubMed and Embase systematic searches with the aid of the CBO employees frequently the Patient-Intervention-Comparison-Outcome (PICO) method was used (Robinson and Dickersin 2002, Poolman et al. 2007a). A foreground question narrows down the possible answers and is more to the point (Petrisor and Bhandari 2006) all four elements were combined and translated into a database search strategy. Methodological search filters proved to be very valuable. The Scottish Intercollegiate Guidelines Network (SIGN) provides practical examples of these filters (www.sign.ac.uk/guidelines/index.html).

### Literature search, assessment selection factors and literature selection

The actual searches with relevant search terms resulted altogether in thousands of hits. Selection factors were, for example, language (English, German, French, or Dutch), no case reports nor letters, meta-analysis, and systematic review. From the records selected, abstracts were evaluated. For records that were relevant, the corresponding papers were retrieved.

### Evaluation of selected literature and evidence table

Assessment of the quality of the literature selected concerned the internal validity, where a judgment was made on selection bias, information bias and confounding. Then an evidence table was constructed for groups of studies concerning one subject, in which following parameters were scored: year of publication, randomization, blinding, set-up, number of patients, duration of treatment, lost to follow-up (number, reason), outcomes, efficacy, safety, and grading of level of evidence. Grading was done according to Grades of Recommendation Assessment, Development, and Evaluation (GRADE) (GRADE Working Group 2004).

### Writing and discussion of concept text

Grading quality of evidence was directly related, but only one factor when determining the strength of recommendations (Schunemann et al. 2006b, Akl et al. 2007, Guyatt et al. 2008a). Each member focused on one or two subjects and was supervised by another colleague. In general, the text of each topic contained an introduction with an explanation of the problem and a summary of the current general literature. Each topic was explained, the relevant literature was discussed, and other considerations were evaluated (sometimes these were non-medical). In the end, a highlighted and boxed recommendation was formulated and graded, again according GRADE (Schunemann et al. 2006b, Guyatt et al. 2008a).

A key element of reporting guidelines is the use of a structured format. Quality of evidence and strength of recommendations should be reported clearly using a standard approach (Oxman et al. 2006).

### Comments on the guideline from users, and authorization procedure

The concept text is now presented to the various users. Depending on the subject, it is possible to invite selected experts to comment, choose a cross-sectional panel, or address all users. The Dutch Orthopaedic Association placed the text on its website and communicated to all its members that they were invited to make comments by e-mail. At the next national assembly, the guideline was approved. This authorization is a formality where generally content and procedure are not discussed but important for its acceptance in clinical practice. There is evidence that wide representation of stakeholders in guideline panels leads to more balanced recommendations. While we have not evaluated this for the example described in this article, we believe that the generalization from studies to our guideline effort is secure. We achieve this wide representation through participation of 23 organizations (250 delegates).

### Implementation of a guideline

Strategies of implementation should be tailored to the intended users, who are health professionals and their organizations. Several models are available for planning and achievement of desired improvements, depending on the type of guideline. Dissemination of the guideline plays a key role in its implementation (Fretheim et al. 2006a). The distribution of the authorized text to the users through a mailing from the various associations and to the hospitals and its administrators and publication in peer reviewed journals and websites, are essential. Also, patient information and flow charts can be valuable. Shiffman and co-workers developed and validated a tool to help in guideline implementation: “The Guideline Implementability Appraisal” (GLIA) (Shiffman et al. 2005).

In order to determine quality in healthcare, there is a need for indicators. Evidence-based guidelines are particularly suited to development of indicators. The requirements for these indicators are:

1. Relevant to clinical practice
2. Related to patientcare
3. Result(s) in improvement
4. Sufficiently specific and sensitive
5. Readily available
6. Wide applicability
7. Relevant to society
8. Strong recommendations, or documentation that a decision-making process has taken place for weak recommendations.

The evaluation of the last 1999 Dutch guideline of the section prevention VTE orthopedic surgery was evaluated through a questionnaire send to all orthopedics departments (Ettema et al. 2005). This is an appraisal of external indicators. In the case of a procedure such as a hip or knee arthroplasty, the use of a specific kind of pharmacological thromboprophylaxis is a process indicator and the incidence of VTE (according to a strict definition) is an outcome indicator.
